# Meiotic and mitotic aneuploidies drive arrest of in vitro fertilized human preimplantation embryos

**DOI:** 10.1186/s13073-023-01231-1

**Published:** 2023-10-02

**Authors:** Rajiv C. McCoy, Michael C. Summers, Abeo McCollin, Christian S. Ottolini, Kamal Ahuja, Alan H. Handyside

**Affiliations:** 1 Department of Biology, Johns Hopkins University, 3400 N. Charles Street, Baltimore, MD 21212 USA; 2 London Women’s Clinic, 113-115 Harley Street, Marylebone, London, W1G 6AP UK; 3 School of Biosciences, University of Kent, Canterbury, CT2 7NJ Kent UK; 4 Present Address: London Women’s Clinic, The Chesterfield, Nuffield Health Clinic, 3 Clifton Hill, Bristol, BS8 1BN UK; 5 Department of Maternal and Fetal Medicine, University College London, 86-96 Chenies Mews, London, WC1E 6HX UK; 6 Present Address: Juno Genetics Italia, Via Di Quarto Peperino 22, 00188 Rome, Italy

**Keywords:** Monosomy, Trisomy, Meiosis, Mitosis, IVF, Preimplantation genetic testing, Time-lapse

## Abstract

**Background:**

The high incidence of aneuploidy in early human development, arising either from errors in meiosis or postzygotic mitosis, is the primary cause of pregnancy loss, miscarriage, and stillbirth following natural conception as well as in vitro fertilization (IVF). Preimplantation genetic testing for aneuploidy (PGT-A) has confirmed the prevalence of meiotic and mitotic aneuploidies among blastocyst-stage IVF embryos that are candidates for transfer. However, only about half of normally fertilized embryos develop to the blastocyst stage in vitro, while the others arrest at cleavage to late morula or early blastocyst stages.

**Methods:**

To achieve a more complete view of the impacts of aneuploidy, we applied low-coverage sequencing-based PGT-A to a large series (*n* = 909) of arrested embryos and trophectoderm biopsies. We then correlated observed aneuploidies with abnormalities of the first two cleavage divisions using time-lapse imaging (*n* = 843).

**Results:**

The combined incidence of meiotic and mitotic aneuploidies was strongly associated with blastocyst morphological grading, with the proportion ranging from 20 to 90% for the highest to lowest grades, respectively. In contrast, the incidence of aneuploidy among arrested embryos was exceptionally high (94%), dominated by mitotic aneuploidies affecting multiple chromosomes. In turn, these mitotic aneuploidies were strongly associated with abnormal cleavage divisions, such that 51% of abnormally dividing embryos possessed mitotic aneuploidies compared to only 23% of normally dividing embryos.

**Conclusions:**

We conclude that the combination of meiotic and mitotic aneuploidies drives arrest of human embryos in vitro, as development increasingly relies on embryonic gene expression at the blastocyst stage.

**Supplementary Information:**

The online version contains supplementary material available at 10.1186/s13073-023-01231-1.

## Background

Following natural conception, many human embryos are chromosomally abnormal and are progressively eliminated through preclinical pregnancy loss, miscarriage, and stillbirth, such that the overall incidence of these abnormalities detected in newborns is less than 1% [1, 2]. Observed chromosome abnormalities include genome-wide abnormalities in ploidy (e.g., triploidy), as well as whole and segmental aneuploidies of individual chromosomes [3]. Whole chromosome aneuploidy frequently arises through errors in meiosis, predominantly in females, resulting in aneuploid oocytes. Risk of such maternal meiotic aneuploidies, particularly of the smaller and acrocentric chromosomes, increases exponentially for women over the age of 35 years in parallel with increasing risk of miscarriage [4]. A similar pattern of aneuploidy is also observed following in vitro fertilization (IVF) [5, 6]. Hence, preimplantation genetic testing for aneuploidy (PGT-A) at the blastocyst stage, by trophectoderm biopsy and next-generation sequencing (NGS)-based chromosome copy number analysis, is widely used to prioritize transfer of embryos with euploid test results [7]. However, the degree to which the biopsy is representative of the rest of the embryo is a matter of ongoing research [8], and the clinical efficacy of PGT-A is the subject of long-standing controversy [9].

Unlike previous methods used for PGT-A, including, for example, array comparative genomic hybridization (aCGH), NGS exhibits a linear relationship between normalized read depth and chromosome copy number [10]. With multiple trophectoderm cells (typically 5–10 cells) biopsied at the blastocyst stage, this has enabled measurement of read depth deviations ranging from those expected for constitutional trisomies or monosomies (i.e., full copy number changes) to those expected for mosaic aneuploidies (i.e., intermediate copy number changes; [11]). By identifying meiotic errors in polar bodies and trophectoderm biopsies using single nucleotide polymorphism (SNP) genotyping and karyomapping in parallel with NGS-based PGT-A, we recently demonstrated that, with one exception, all female meiotic aneuploidies produced read depth deviations exceeding 70% of that expected of a full copy number change [12]. In contrast, most non-meiotic (presumed mitotic origin) aneuploidies had read depth deviations ranging from 30 to 70% that expected a full copy number change, although a minority exceeded the 70% threshold and may have resulted from chromosome missegregation in the first mitotic cleavage division.

Despite improvements in embryo culture, only about half of normally fertilized embryos reach the blastocyst stage, while the remainder arrest at various cleavage, late morula, or early blastocyst stages [13–16]. As early as 1993, Munné and colleagues demonstrated the association between aneuploidy, asymmetric cleavage, and embryo arrest using multicolor interphase fluorescence in situ hybridization (FISH) with chromosome-specific probes for X, Y, and 18 [17]. Since then, with the introduction of comprehensive chromosome testing methods, numerous studies have confirmed this association [18–21]. However, all of these studies were limited by the use of earlier methodologies that did not discriminate between full and intermediate copy number changes for all chromosomes, limited sampling of embryo cells, or analysis of selected clinical-grade embryos only (reviewed in [22]).

Here, we use the established copy number thresholds [12] to discriminate meiotic- and mitotic-origin aneuploidies based on NGS of a large sample of arrested embryos and trophectoderm biopsies of blastocysts irrespective of their morphological grade. We note that our study is not designed to evaluate the clinical efficacy of PGT-A and therefore should not be read as an endorsement of its use or non-use. Rather, our study applies the tools developed for PGT-A to address the relationship between aneuploidy and preimplantation embryo arrest, within technical and ethical limits. By testing both arrested embryos and blastocysts from the same IVF cycles, we infer the relative contributions of various forms of aneuploidy to preimplantation embryo arrest. Furthermore, as morphokinetic parameters are reported to be altered in aneuploid embryos [23–25], time-lapse analysis was used to identify abnormalities in the first and/or second cleavage divisions and correlate these with aneuploidy and developmental outcomes. Finally, by mitigating the survivorship biases that affect most retrospective studies, our study refines estimates of the incidences of meiotic and mitotic aneuploidy and their relationships with maternal age. Together our work offers a detailed view of chromosome and cleavage abnormalities in human preimplantation embryos and their contributions to embryonic mortality.

## Methods

### Study design and informed consent

This report represents data from a subset of patients that were part of a prospective cohort single-center study of IVF with blastocyst vitrification-only and optional PGT-A (VeriSeqPGS, Illumina, USA) between January 2016 to June 2018, as previously described by Gorodeckaja et al. [26]. Specifically, this subset of patients signed the HFEA Consent to Disclosure form for either contact or non-contact research based on their data. Together, this cohort includes 125 patients (mean 38.9 years at oocyte retrieval; range 30–45 years) who underwent a total of 165 IVF cycles with extended embryo culture to the blastocyst stage and biopsy of 5–10 trophectoderm cells on days 5–7 post-insemination for PGT-A. An additional 22 cycles in which all embryos arrested and were not tested by PGT-A (a total of 52 zygotes with two pronuclei [2PN]) were excluded from further analysis.

### Time-lapse image analysis and blastocyst grading

Time-lapse incubation was used to maintain an uninterrupted embryo culture environment. Embryo development was monitored continuously up to seven days post insemination, or until blastocyst formation and expansion, if earlier, by time-lapse imaging and software analysis (Geri® Connect, GeneaBiomedx, Sydney, Australia). Blastocysts were evaluated by assigning a letter grade (A through D) to the inner cell mass (ICM) and trophectoderm (TE) based on standardized morphological criteria (Additional file 1: Fig. S1; Additional file 2: Table S1) [27]. Grading of top-quality blastocysts was more restrictive, whereas very poor-quality blastocysts were given a D grade based on very few cells in the ICM and TE and evidence of cellular degeneration. Time-lapse videos of all embryos were annotated daily using the manufacturer’s software and documentated in the patient’s laboratory records. Assessment included several time points in the development of each embryo: (1) formation of pronuclei, (2) first cleavage division, (3) second cleavage division, (4) cell compaction, (5) cavitation, (6) expanded blastocyst, or (7) embryo arrest image prior to processing embryos for PGT-A. The division pattern was recorded for each embryo as per Ottolini et al. [28] and based on the number of cells after each division to indicate either normal (i.e., 1 → 2 → 4 cells) or abnormal (e.g., 1 → 3 → 6 cells) cleavage patterns [29–32]. Categories of abnormal division included multipolar, precocious, reverse, and failed cleavage [33]. “Multipolar” cleavage refers to the direct cleavage of the zygote (or a daughter cell) into three or more cells. “Precocious” cleavage refers to a rapid division pattern where the zygote (or a daughter cell) undergoes a normal 1 → 2 cell cleavage, followed by a subsequent premature division to produce 3 or more blastomeres. “Reverse” cleavage refers to the resorption of blastomeres after cytokinesis. “Failed” cleavage refers to multiple rounds of karyokinesis without cytokinesis.

### Biopsy and sampling of embryos

Embryos were cultured to the blastocyst stage, and surviving embryos underwent biopsy of 5–10 trophectoderm cells on days 5–7 post-insemination for clinical purposes. Biopsy samples were washed in Dulbecco’s phosphate buffered saline (DPBS; Gibco; Life Technologies, USA) with 0.1% polyvinyl alcohol (PVA; Sigma-Aldrich, USA) and transferred into PCR tubes (Corning, Sigma-Aldrich, USA) containing 2 μL DPBS and stored at −20° C prior to analysis.

Embryos that showed no evidence of further development by either cell count or compaction and failed to develop to the expanded blastocyst stage by day 7 or showed signs of degeneration were considered arrested in development if no change was seen by time-lapse analysis for at least the preceding 24 h. Arrested embryos were scored as either early, mid, or late as follows: early arrest, 1 cell through 6–10 cells spanning post-fertilization through embryonic genome activation; mid arrest, > 10 cells through pre-compaction; and late arrest, evidence of compaction through early blastocoel formation. The zona pellucida of each arrested embryo was first thinned by brief exposure to acidified Tyrode’s solution (Origio, USA) and removed by gentle pipetting, ensuring the integrity of the entire intact cell mass for subsequent genetic analysis. All selected arrested embryos were then washed and prepared for genetic analysis according to the same steps described above for clinical TE biopsy samples.

DNA from all TE biopsy samples and arrested embryos was whole-genome amplified (WGA) using a polymerase chain reaction (PCR)-library-based method (SurePlex; Illumina, USA), with library preparation and Illumina sequencing performed by Genesis Genetics (Genesis-24, UK).

### Data processing, statistical analysis, and visualization

Raw sequencing data were processed and visualized using Bluefuse Multi software (Illumina, USA). A total of 30 samples (3.2%; 18 arrested embryos and 12 TE biopsies) were excluded from downstream analysis as they failed to meet quality control standards (lack of DNA, excessive technical noise, or evidence of contamination based on negative controls).

Intra-sample chromosome mosaicism is diagnostic of mitotic error and is expected to produce copy number results intermediate between those expected of uniform trisomies or monosomies (full copy number changes) and disomy. In the current study, we used the range of 30-70% of that which is expected for constitutional aneuploidy (i.e., affecting all cells) to define the thresholds for calling mitotic-origin (i.e., mosaic) aneuploidy. Values above 70% were considered as meiotic aneuploidies, whereas values below 30% were considered as normal disomies.

In previous work, we extensively validated these thresholds by parallel analysis of polar bodies and embryo samples by SNP genotyping and karyomapping [12]. Our results showed that the NGS-based copy number exhibited concordance with SNP classification in 86% of TE biopsy samples, as well as 75% of whole or partial arrested embryo samples. While these benchmarking results imply that a small proportion of aneuploidies (< 25%) are likely mis-classified in the current study, we emphasize that the same thresholds were applied across all samples, thereby ensuring the robustness of comparisons between embryos at different stages or with different developmental outcomes. We also acknowledge that precise copy number displacements are less reliable when multiple chromosomes are simultaneously affected by aneuploidy, though this limitation again equally affects all categories of embryos. Examples of copy number plots are provided in Additional file 1: Fig. S2.

Sex chromosome aneuploidies and segmental aneuploidies were not stratified into meiotic and mitotic aneuploidy categories, because the probability of mis-classification is much higher for these groups. In the case of sex chromosome aneuploidies, the baseline copy number expectations depend on the assumed sex of the embryo, which itself must be inferred. In the case of segmental aneuploidies, the smaller affected regions result in a lower ratio of signal to noise in normalized read depth. Moreover, individual genomic bins may encompass copy number variant (CNV) breakpoints, causing the erroneous detection of intermediate copy number segments.

All statistical analysis was conducted in R (version 4.1.2). Figures were produced using the “ggplot2” [34] and “ggsankey” [35] packages.

The relationships between the probability of embryo arrest and various predictor variables (e.g., Fig. 4) were modeled using binomial generalized linear mixed effects models (GLMMs), implemented with the “GLMMadaptive” package [36]. The patient identifier was included as a random effect in all models, thereby addressing the non-independence among sets of embryos sampled from the same patient. Specifically, in the first model, the response variable was a binary indicator denoting whether the embryo arrested, while the copy number result category was the sole categorical predictor variable. The second model used the same response variable, but the total number of whole aneuploid autosomes (meiotic or mitotic) was the sole numeric predictor variable. The third model again used the same response variable, but the number of cells after the first mitotic division was included as the sole categorical predictor variable, where the possible categories were 1, 2, 3, 4, and > 4. In all cases, coefficient estimates and 95% confidence intervals were converted from the logit to the probability scale to facilitate interpretation. As coefficient estimates from GLMMs are conditional on the level of the random effect (i.e., patient), we report the average marginal effect (AME), which averages over all levels of the random effect.

The relationship between aneuploidy and the day of blastocyst biopsy was modeled using a linear mixed effects model (LMM), implemented with the “lme4” package [37], with AMEs computed using the “margins” package [38].

Key results were replicated upon restricting analysis to the subset of IVF cases where all embryos were tested with PGT-A (see Additional file 1: Supplementary Materials).

## Results

**Fig. 1:**
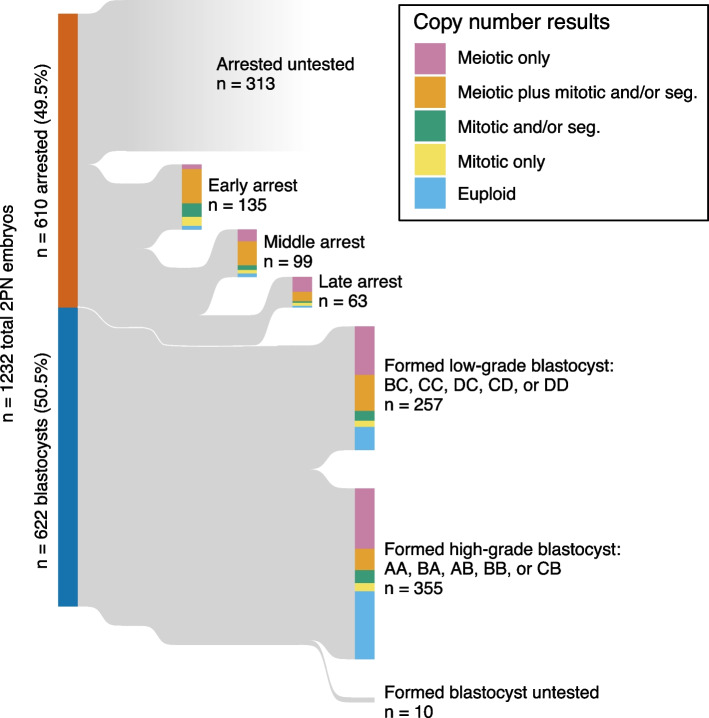
Developmental outcomes of 1232 normally fertilized (2PN) embryos and their associated PGT-A results. A total of 909 (73.8%) of embryos were tested with PGT-A, including 297 of 610 (48.7%) arrested embryos and 612 of 622 (98.4%) blastocysts. Early arrest: cleavage stages; ≤ 10 cells. Mid arrest: > 10 cells, but pre-compact morula. Late arrest: compact morula to cavitating (non-expanded) blastocyst

Among the 165 IVF cycles (125 unique patients) included in our study (see the “Methods” section), an average of 3.8 of 7.2 (53%) embryos per cycle reached the blastocyst stage. This proportion was negatively associated with maternal age (quasi-binomial generalized lienar model [GLM]: coefficient estimate [$\widehat\beta$] = −0.080, standard error [SE] = 0.022, *p* value [*p*] = 3.7 × 10^−4^; Additional file 2: Table S2). Considering all 1232 normally fertilized (2PN) zygotes and excluding possible triploid embryos subsequently identified by PGT-A (see the “Methods” section), 622 (50.5%) embryos developed to the blastocyst stage and 610 (49.5%) embryos arrested between the zygote and late morula/early blastocyst stages (Fig. 1). A total of 909 (73.4%) embryos derived from 2PN zygotes were tested with PGT-A, including 612 of the 622 (98.4%) blastocysts and 297 of the 610 (48.7%) arrested embryos. Notably, this includes 85 (51.5%) cycles in which all embryos were tested, as well as 80 cycles (48.5%) in which only a subset (mean = 24.1%) of arrested embryos were tested. While we opted to analyze all 165 cycles to maximize statistical power, we also replicated key results in the subset of 85 cycles where all embryos were tested, as presented in the supplementary materials. We highlight the investigation of arrested embryos as well as poorer quality blastocysts (257 embryos with morphological grades BC, CC, CD, DC, or DD) as important aspects of our study, as such embryos are not frequently tested in clinical settings because they are not typically considered as good candidates for either biopsy, vitrification, or transfer.

### Meiotic and mitotic aneuploidies are prevalent among preimplantation embryos

Across 909 tested embryos, 206 (22.6%) were euploid while 703 (77.3%) possessed whole or segmental aneuploidies of one or more chromosomes (Fig. 1). To gain insight into the origins of aneuploidies and their consequences for development, we distinguished putative meiotic and mitotic aneuploidies based on their PGT-A copy number profiles (see the “Methods” section). Along with 50 aneuploidies of entire sex chromosomes, for which discerning mosaic aneuploidy poses unique challenges (see the “Methods” section), we discovered 1154 putative meiotic and 1051 putative mitotic aneuploidies of whole autosomes. We additionally identified 358 large segmental aneuploidies (on the scale of entire chromosome arms), including 351 affecting autosomes and 7 affecting sex chromosomes.

**Fig. 2:**
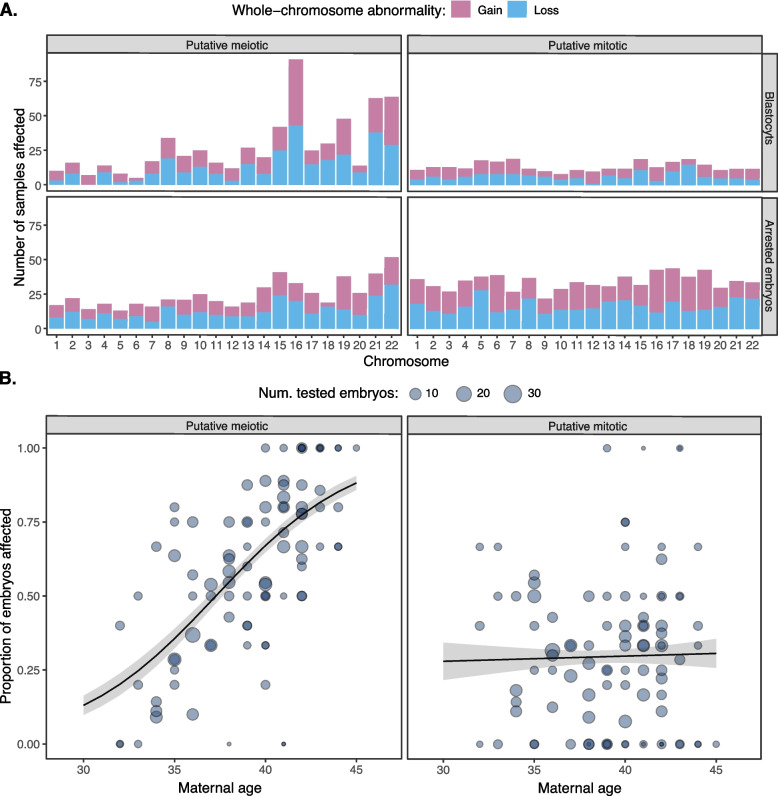
Chromosome-specific rates and age associations for putative meiotic- and mitotic-origin aneuploidies.** A** Chromosome-specific counts of putative meiotic (i.e., full copy number change; left panel) and putative mitotic (i.e., intermediate copy number change; right panel) whole-chromosome gains and losses observed in blastocysts (top panel) and arrested embryos (bottom panel) as determined with PGT-A. Only aneuploidies of entire autosomes are depicted, as distinguishing meiotic and mitotic origins of segmental aneuploidies and aneuploidies affecting sex chromosomes poses unique challenges (see the “Methods” section). **B** Observed rates of meiotic (left panel) and mitotic (right panel) aneuploidy in relation to maternal age, including both arrested embryos and blastocysts. Each data point represents a distinct IVF case. Lines represent predictions from binomial generalized linear models fit to the data, with standard errors of the predictions indicated in gray

Consistent with previous work (e.g., [39]), the meiotic aneuploidies disproportionately impacted chromosomes 15, 16, 19, 21, and 22, whereas mitotic aneuploidies exhibited similar frequencies across all autosomes (Fig. 2A; Additional file 1: Fig. S3). Moreover, we confirmed that the putative meiotic aneuploidies possessed a strong association with maternal age (quasi-binomial GLM: $\widehat\beta$ = 0.261, SE = 0.032, *p* = 1.68 × 10^−12^; Fig. 2B), whereas putative mitotic aneuploidies exhibited no significant age association (quasi-binomial GLM: $\widehat\beta$ = 0.009, SE = 0.033, *p* = 0.799; Fig. 2B).

We observed no significant co-occurrence of meiotic and mitotic aneuploidies affecting different chromosomes of the same embryos (Fisher’s exact test: odds ratio [OR] = 1.32, 95% confidence interval [CI; 0.99, 1.77], *p* = 0.055; Additional file 2: Table S3), suggesting that these error mechanisms are largely independent. However, when restricting to the 612 blastocyst-stage embryos, we observed a modest enrichment of mitotic aneuploidy among embryos already affected by meiotic aneuploidy (Fisher’s exact test: OR = 1.54, 95% CI [1.02, 2.36], *p* = 0.036; Additional file 2: Table S4). While the wide confidence intervals suggest that our power for detecting such an effect is limited in both cases, the latter observation raises the intriguing hypothesis that following embryonic genome activation, the functional effects of meiotic aneuploidies may include impacts on the mitotic machinery, compromising the fidelity of chromosome segregation.

### Mitotic aneuploidies disproportionately contribute to preimplantation arrest

The overall incidence of aneuploidy among arrested embryos was 94%, compared to 69% in tested blastocysts (Fig. 1). Both putative meiotic and putative mitotic whole-chromosome aneuploidies were enriched among arrested embryos compared to embryos that developed to the blastocyst stage, though the effect was much stronger for mitotic aneuploidies (Fisher’s exact test: OR = 6.02, 95% CI [4.40, 8.28], *p* = 4.22 × 10^−33^; Additional file 2: Table S5) compared to meiotic aneuploidies (Fisher’s exact test: OR = 1.62, 95% CI [1.20, 2.20], *p* = 0.0012; Additional file 2: Table S6).

**Fig. 3:**
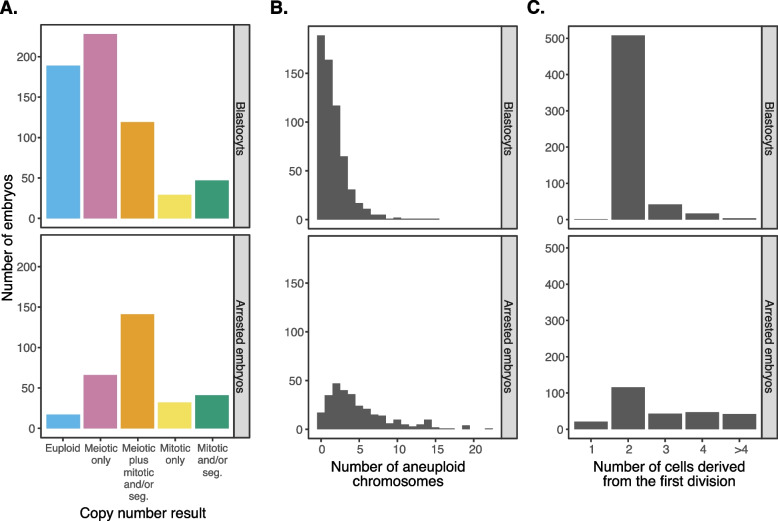
Contrasting chromosomal and cellular characteristics of arrested embryos with developing blastocysts. **A** Counts of arrested embryos versus developing blastocysts, stratified by PGT-A copy number result category. **B** Counts of arrested embryos versus developing blastocysts, stratified by the total number of aneuploid chromosomes. **C** Counts of arrested embryos versus developing blastocysts, stratified by the number of cells present after the first mitotic division

For downstream analysis and visualization, we further grouped embryos into five mutually exclusive categories: (1) euploid, (2) meiotic whole-chromosome aneuploidy only, (3) meiotic whole-chromosome aneuploidy in combination with mitotic whole-chromosome aneuploidy or segmental aneuploidy, (4) mitotic whole-chromosome aneuploidy only, and (5) segmental aneuploidy with or without mitotic whole-chromosome aneuploidy (Table 1). The distribution of embryos among these categories was significantly different for arrested embryos compared to trophectoderm biopsies of developing blastocysts (Fig. 3A; Pearson’s chi-squared test: *χ*^2^ [4, *N* = 909] = 143.4, *p* = 5.41 × 10^−30^; Additional file 1: Fig. S4A).

**Table 1:** Incidence of various forms of aneuploidy in whole arrested embryos (see the “Methods” section for description of arrested embryos) versus trophectoderm biopsies of developing blastocysts as determined by preimplantation genetic testing for aneuploidy (PGT-A). Our approach for distinguishing putative meiotic and mitotic aneuploidies based on chromosome copy number results is detailed in the main text

Putative chromosome status	Abbreviation	Whole arrested embryos	Blastocyst-stage embryos (trophectoderm biopsies)
Euploid	Euploid	17 (6%)	189 (31%)
Meiotic whole-chromosome aneuploidy only	Meiotic only	66 (22%)	228 (37%)
Meiotic whole-chromosome aneuploidy with mitotic whole-chromosome aneuploidy or segmental aneuploidy	Meiotic plus mitotic and/or seg	141 (47%)	119 (19%)
Mitotic whole-chromosome aneuploidy only	Mitotic only	32 (11%)	29 (5%)
Segmental aneuploidy with or without mitotic whole-chromosome aneuploidy	Mitotic and/or seg	41 (14%)	47 (8%)
**Total**		297	612

**Fig. 4:**
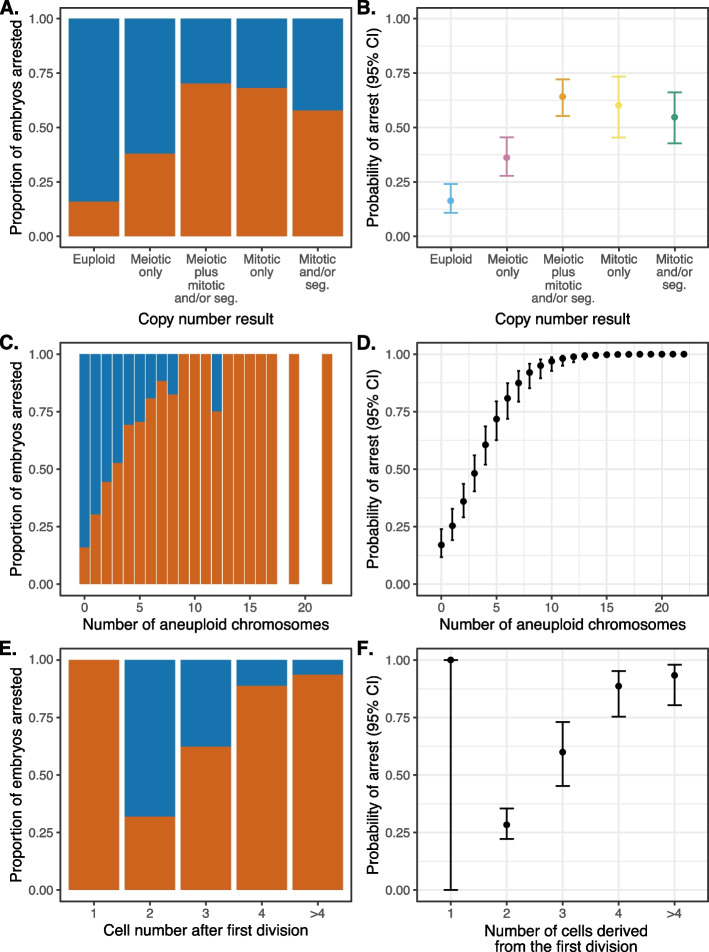
Data (left panels) and statistical modeling (right panels) of the proportion/probability of embryo arrest, stratifying on various patterns of chromosome copy number or cell division. Error bars denote 95% confidence intervals of estimates. **A** Proportion of arrested (red) versus unarrested (blue) embryos, stratifying on chromosome copy number pattern, as assessed by PGT-A. **B** Statistical modeling of the data from panel **A**. **C.** Proportion of arrested (red) versus unarrested (blue) embryos, stratifying on the number of aneuploid chromosomes. **D** Statistical modeling of data from panel **C**.** E** Proportion of arrested (red) versus unarrested (blue) embryos, stratifying on the number of cells observed after the first mitotic division, where 2 is normal. **F** Statistical modeling of data from panel **E**

Whereas euploid embryos exhibited a 16% frequency of arrest (95% CI [10%, 25%]), embryos with solely meiotic aneuploidy (“Meiotic only”) arrested at a 36% frequency (95% CI [28%, 45%]), while embryos with mitotic aneuploidies (“Meiotic plus mitotic and/or segmental,” “Mitotic only,” “Mitotic and/or segmental”) arrested at much higher frequencies (ranging from 55 to 64%; Fig. 4A, B; Additional file 1: Fig. S5A, B). Moreover, the number of aneuploid chromosomes per embryo was strongly associated with the frequency of arrest, with complex aneuploidies arresting in much higher proportions (binomial generalized linear mixed model [GLMM]: average marginal effect [AME] = 0.440, SE = 0.054, *p* = 4.04 × 10^−16^; Figs. 3B and 4C, D; Additional file 1: Fig. S4B; Additional file 1: Fig. S5C, D), building upon previous observations from smaller samples [40].

The higher rate of arrest among embryos with mitotic compared to meiotic aneuploidies is potentially counterintuitive, as the former affects only a fraction of cells whereas the latter affects all cells of the embryo. This paradox may be explained, however, by the fact that mitotic aneuploidies affected more chromosomes than meiotic aneuploidies, on average per embryo (quasi-Poisson GLM: $\widehat\beta$ = 0.418, SE = 0.055, *p* = 4.6 × 10^−14^; Additional file 1: Fig. S6). Indeed, after accounting for the number of chromosomes affected (i.e., the complexity of aneuploidy), the form of aneuploidy (i.e., meiotic versus mitotic) had no significant effect on probability of embryo arrest (likelihood ratio test statistic = 6.08, *p* = 0.108). Similarly, maternal age was not a significant predictor of embryo arrest after accounting for the form of aneuploidy (i.e., meiotic versus mitotic; likelihood ratio test statistic = 0.57, *p* = 0.452) or number of chromosomes affected (likelihood ratio test statistic = 2.17, *p* = 0.141), suggesting that the maternal age effect on IVF embryo loss is nearly exclusively mediated by aneuploidy.

Given the strong association between aneuploidy and pregnancy loss, one quantity of fundamental interest is the proportion of embryos derived from euploid zygotes that arrest during preimplantation development, as these compose the initial pool of potentially viable zygotes whose success we seek to maximize. The ability to estimate this parameter has been hindered by the fact that early-arresting embryos are not typically tested in clinical settings. Within our data, the embryos derived from euploid zygotes include any embryo that lacks evidence of meiotic aneuploidy. Fitting a GLMM to our data (Additional file 1: Fig. S7A; see the “Methods” section), we found that embryos in this group arrested with a probability of 38% (95% CI [31%, 45%]; Additional file 1: Fig. S7B), whereas embryos derived from the remaining aneuploid zygotes arrested with a probability of 50% (95% CI [44%, 57%]; Additional file 1: Fig. S7B).

### Abnormal cell divisions drive lethal mitotic aneuploidies

To gain insight into the relationship between abnormal early cell divisions and mitotic aneuploidies, we used time-lapse imaging (see the “Methods” section) to record the first two cell divisions of 843 embryos derived from 2PN zygotes following fertilization. Overall, 219 embryos (26.0%) exhibited an abnormal first division, while an additional 82 embryos (9.7%) exhibited an abnormal second division (Additional file 2: Table S7). The former group includes 85 embryos (10.1%) that underwent abnormal division from one into three cells, largely due to precocious or multipolar cell divisions. We observed a strong association between abnormal cell division and putative mitotic aneuploidy, with 51% of abnormally dividing embryos possessing mitotic aneuploidies compared to only 23% of normally dividing embryos (Fisher’s exact test: OR = 3.41, 95% CI [2.50, 4.67], *p* = 6.93 × 10^−16^; Additional file 2: Table S8). No such relationship was observed between abnormal cell division and meiotic aneuploidy (Fisher’s exact test: OR = 1.20, 95% CI [0.89, 1.62], *p* = 0.217; Additional file 2: Table S9). As further expected, abnormal cell divisions were also strongly associated with embryonic arrest (Fisher’s exact test: OR = 10.86, 95% CI [7.67, 15.50], *p* = 2.72 × 10^−50^; Additional file 2: Table S10; Fig. 3C; Additional file 1: Fig. S4C; Fig. 4E, F; Additional file 1: Fig. S5E, F).

Consistent with this interpretation, the probability that embryos derived from euploid zygotes would arrest strongly depended on the outcomes of the first and second cell divisions. Specifically, the probability that an embryo derived from a euploid zygote would arrest was 12% (95% CI [7%, 19%]) when conditioning on normal first and second mitotic divisions compared to 34% (95% CI [27%, 42%]) for aneuploid zygotes (Additional file 1: Fig. S8A, B). These proportions increased to 75% (95% CI [62%, 84%]) for euploid zygotes as well as 75% (95% CI [66%, 82%]) for aneuploid zygotes when conditioning on abnormal first or second cell divisions (Additional file 1: Fig. S8C, D).

Notably, even embryos lacking evidence of meiotic or mitotic aneuploidies were much more likely to arrest if they experienced an abnormal first or second cell division (Fisher’s exact test: OR = 19.90, 95% CI [5.59, 90.50], *p* = 9.12 × 10^−8^; Additional file 2: Table S11). Though this accounts for only 13 embryos in total (compared to 4 euploid embryos that arrested following normal cell divisions), the observed enrichment suggests that the fitness impacts of abnormal cell division are not solely attributable to aneuploidy or that certain forms of mosaic aneuploidy could be masked by bulk DNA analysis (see the “Discussion” section).

### Aneuploidy is strongly associated with blastocyst morphology

**Fig. 5:**
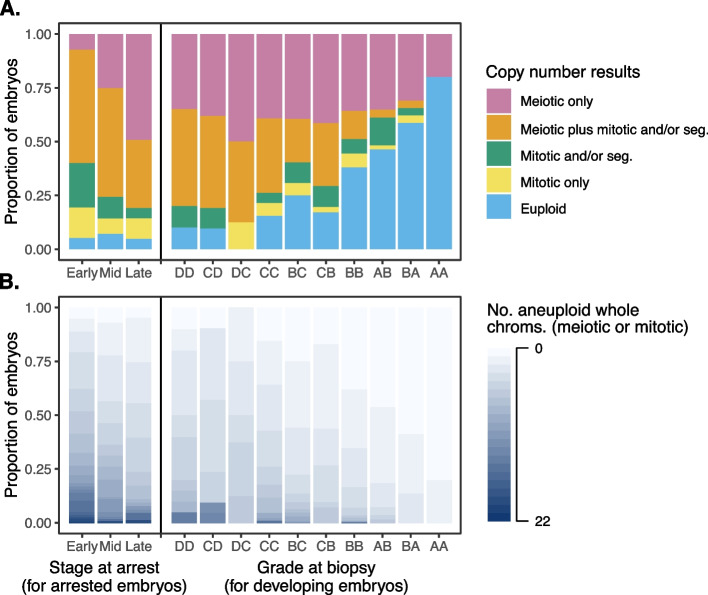
Chromosome copy number results (as assessed via PGT-A) across all tested embryos, stratifying by stage at arrest (see the “Methods” section for description of arrested embryos) or morphological grade (for embryos that formed blastocysts). ICM grade is listed first, and TE grade is listed second. **A** Copy number results assigned to categories, as described in Table 1. **B** Copy number results summarized as counts of aneuploid chromosomes

Among embryos that survived to the blastocyst stage, we sought to understand the relationship between various forms of aneuploidy and blastocyst morphology, with the rationale that aneuploidies may compromise the organization and function of the differentiating cell lineages. Blastocysts were graded by assigning an ordinal letter grade (A through D) to the inner cell mass as well as the trophectoderm based on standardized morphological criteria (Additional file 1: Fig. S1; Additional file 2: Table S1) [27]. Notably, morphological grading was conducted at the time of embryo culture, prior to obtaining and thereby blind to the PGT-A results. Within our sample, the grades for the inner cell mass and trophectoderm were strongly correlated, consistent with the interpretation that ploidy status and other shared genetic and environmental factors simultaneously impact both cell types (Additional file 1: Fig. S9). We observed a strong association between aneuploidy status and blastocyst morphology, with aneuploidy rates ranging from 20 to 90% for embryos with the highest to lowest grades. Poor morphology embryos were significantly enriched for aneuploidy (Pearson’s chi-squared test: *χ*^2^ [9, *N* = 612] = 60.2, *p* = 1.23 × 10^−9^; Fig. 5; Additional file 1: Fig. S10). This relationship was consistent for embryos affected by meiotic aneuploidies (Pearson’s chi-squared test: *χ*^2^ [9, *N* = 612] = 49.4, *p* = 1.38 × 10^−7^) as well as those affected with mitotic aneuploidies (Pearson’s chi-squared test: *χ*^2^ [9, *N* = 612] = 49.3, *p* = 1.45 × 10^−7^) when compared to euploid embryos. Additionally, the presence of meiotic and/or mitotic aneuploidies was associated with later timing of blastocyst biopsy, indicating that aneuploidies tend to delay the process of blastocyst formation and expansion (linear mixed model [LMM]: AME = 0.220, SE = 0.052, *p* = 2.4 × 10^−5^; Additional file 1: Fig. S11).

## Discussion

Here we applied a combination of genome-wide NGS-based copy number analysis and time-lapse imaging to investigate a large sample of both arrested and developing embryos, irrespective of their morphological grade or any other criteria, toward a more comprehensive view of the impacts of chromosome and cell division abnormalities on human preimplantation development. By comparing patterns of putative meiotic and mitotic aneuploidies across these samples and stages, we observed that all categories of aneuploidy were enriched in arrested embryos versus developing blastocysts, reflecting their respective contributions to embryonic mortality. The leading cause of IVF embryo arrest appears to be lethal mitotic aneuploidies, which are in turn associated with abnormal first or second mitotic divisions and tend to affect multiple chromosomes simultaneously.

We note that the overall rates of aneuploidy observed in our data (77.3%) are higher than in some previous studies (e.g., [41, 42]). This observation is explained by the age distribution of the patient cohort, as well as the fact that most previous studies were based on retrospective analysis of data from embryos that were candidates for IVF transfer, systematically enriched for surviving embryos with good morphology and thus lower rates of both meiotic and mitotic aneuploidy. By mitigating these biases, our data thus provide valuable fundamental estimates of the underlying rates of meiotic and mitotic aneuploidy in human zygotes and early embryos.

Previous work has demonstrated that the first mitotic division is especially prone to errors, potentially due to the challenge of assembling and segregating chromosomes on a dual mitotic spindle, whereby the maternally and paternally inherited genomes cluster, but remain separate [43–46]. Errors in this process are associated with erroneous attachments between microtubules and kinetochores and/or multipolar chromosome segregation. Our data support that such mitotic aneuploidies are prevalent and occur at similar frequencies for embryos derived from zygotes affected or unaffected with prior meiotic aneuploidies, in turn suggesting an intrinsic instability of cleavage-stage development and/or detrimental effects of embryo culture. It has been proposed, for example, that the blastomeres of early cleavage-stage human embryos function autonomously due to the absence of gap junctions and are particularly sensitive to environmental and metabolic perturbations [47]. By contrast, genome integrity at later stages of development may be more robust, following embryonic genome activation and the development of a transporting epithelium at the time of compaction.

Our own work and that of others showed that early chaotic aneuploid cells in embryos that do not arrest are often excluded at the time of compaction [25, 28]. Nevertheless, mosaic aneuploidies, typically affecting one or few chromosomes, are detected in a smaller proportion of blastocyst-stage embryos by PGT-A and are potentially compatible with healthy birth [48] (though see [49] for how this may depend on the features of mosaicism), posing a dilemma regarding their clinical management. While numerous forms of pathogenic mosaic aneuploidy and structural variation have been identified in clinical studies [50], current evidence suggests that mosaic aneuploidies identified in blastocyst-stage embryos are rarely detected at later stages of pregnancy or at birth, though exceptions have been reported [51, 52]. This observation is consistent with a model of selection against aneuploid cells within mosaic embryos during peri- and post-implantation development, as supported by data from mouse and human embryo models [53–55], as well as analyses of single-cell genomic data from human embryos [56, 57]. Interestingly, a recent retrospective study comparing the clinical outcome of single euploid blastocyst transfers with either normal or abnormal early cleavage patterns confirmed a lower implantation rate, clinical and live birth rate for the latter group [58], potentially reflecting mosaicism that was undetected based on a single-trophectoderm biopsy and motivating future work to test this hypothesis. Our long-standing policy has been to de-prioritize blastocysts with early abnormal cleavage patterns with or without PGT-A [26].

The chromosome instability (CIN) and mitotic aneuploidy observed in early cleavage-stage embryos may reflect defects in DNA repair, cell cycle progression, and intracellular signaling because of underlying imbalances in cell regulatory networks, metabolic flux, and cellular dynamics [59–62]. For example, RNA sequencing results of aneuploid cells from early human embryos and blastocysts exhibit transcriptional profiles consistent with DNA damage [24, 63], as well as changes or disruption of cell proliferation [56, 64], p53 signaling, autophagy, and apoptosis [63–66]. Interestingly, recent work by Palmerola et al. [67] confirmed a high level of incomplete DNA replication resulting in chromosome breaks and mitotic aneuploidy in human zygotes, referred to as replication stress. It is tempting to conclude that our findings are consistent with those of Palmerola et al. [67]*,* with the caveat that their studies involved the use of donated vitrified metaphase II (MII) oocytes (see also [68]).

Studies in model systems have demonstrated that aneuploidy compromises cellular fitness [69, 70]. Direct dosage effects of aneuploidy are known to exert proteotoxic and energy stress on cells, increased rates of mutation, and increased rates of chromosome mis-segregation, which may prove lethal during this critical early developmental transition. Excess or depletion of proteins encoded on the aneuploid chromosomes causes stoichiometric imbalances among components of multiprotein complexes, inducing the formation of toxic protein aggregates [71–73]. Recent work in aneuploid yeast suggests that these free proteins and protein aggregates increase the solute concentration within cells, causing chronic hypo-osmotic stress that impairs endocytosis [74].

A small fraction of euploid embryos, ostensibly lacking both meiotic and mitotic aneuploidies, were present among the group of arrested embryos. Our modeling indicates that such euploid embryos arrest with a probability of 16%, in contrast to embryos with meiotic and mitotic aneuploidies, which arrest at much higher frequencies (36% and 55–64%, respectively). The causes of such euploid embryo arrest remain unknown, but likely include genetic factors such as lethal point mutations [75], structural variation [76], or even certain forms of aneuploidy/polyploidy that lie beyond the limits of detection (see later the “Discussion” section), as well as stochastic and environmental factors which may or may not be specific to IVF and embryo culture.

It is perhaps worth noting that human embryo culture media provide only a partial representation (including a protein source, simple salts, energy substrates, and amino acids) of the natural environment to which embryos are exposed in vivo [77]. Consequently, stresses are invariably imposed on preimplantation embryos grown in chemically defined media. Human embryos appear robust and can develop in a wide selection of commercial media, indicating that they compensate and/or adapt to the imposed stresses [13], but may ultimately succumb under extreme conditions [78, 79]. For example, our data demonstrate that approximately one third of euploid zygotes are lost through abnormal cell divisions and/or mitotic errors after being placed in embryo culture medium. The precise environmental tipping points and genetic factors that influence the probability of human embryo arrest merit detailed investigation.

Low-coverage whole-genome NGS-based approaches for PGT-A, such as those employed in our study, can in principle distinguish meiotic and mitotic aneuploidies based on integer versus fractional variation in normalized read depth compared to diploid expectations. However, accuracy is limited by the sampling process of embryo biopsy, as well as technical noise arising from amplification, sequencing, and mapping biases. Our previous work used an independent and validated SNP genotyping approach to benchmark NGS-based classification for arrested embryos and trophectoderm biopsies, using the same platform and thresholds employed in the current study (see the “Methods” section) [12]. Ground truth detection of meiotic aneuploidies was established by analysis of polar bodies. This work demonstrated that sequencing-based copy number analysis correctly identified meiotic aneuploidies based on signatures of full copy number changes in 13 of 13 (100%) of trophectoderm biopsies, while correctly identifying putative mitotic aneuploidies in 11 of 15 (73%) cases—an overall concordance rate of 86%. Results were qualitatively similar for whole or partial arrested embryo samples, which exhibited concordance with polar body data for 65 of 87 embryos (75%). Together, these benchmarking results engender confidence in the current study, which is further bolstered by our replication of known chromosome-specific and age associations observed for putative meiotic but not mitotic aneuploidies.

A second limitation of our study regards the extraction of bulk DNA from multi-cell (~ 5–10 cells) TE biopsies as well as entire arrested embryos, such that the signatures obtained from sequencing represent an average across cells. While many forms of aneuploidy are readily distinguished, certain patterns, such as polyploidy and balanced cellular representation of monosomies and trisomies (e.g., arising by mitotic non-disjunction) could be missed entirely. In addition, the data did not enable inference of the parent of origin of aneuploidies. Future work in this area may employ disaggregation of individual cells as well as karyomapping or other haplotype-based approaches to trace the transmission of specific homologs across cell divisions and reveal haplotype-based signatures of meiotic error [12, 28, 80]. Haplotype-based methods may also facilitate the analysis of sex chromosomes and smaller segmental aneuploidies, which could not be accurately classified as meiotic or mitotic in origin using the current NGS-based platform. While improving sensitivity, embryo disaggregation and single-cell approaches are not without drawbacks, as numerous cells are lost during library preparation, while the costs and labor-intensive nature of the assay place practical limits on sample size, which was a strength of the current study. Indeed, our previous study used karyomapping to investigate single cells disaggregated from a smaller sample of arrested embryos (*n* = 25), as well as TE biopsies (*n* = 26) and cells excluded from developing blastocysts (*n* = 7), revealing both meiotic and mitotic aneuploidies [28]. Clones of hypodiploid cells with multiple monosomies and nullisomies were identified as would be expected after tripolar mitosis, which was confirmed by time-lapse imaging. Furthermore, complementary sets of parental chromosomes were present in the three clonal lineages in several examples, confirming that the zygote was euploid. Theoretically, if the number of descendants in each clone was equal, the NGS of the whole embryo would not detect mosaicism. However, in each such case, there were varying proportions of clonal descendants and/or near-diploid cells, such that multiple mosaic aneuploidies would be detected by bulk analysis, as observed for several embryos in the current study.

## Conclusions

In their provocatively titled article, “Where have all the conceptions gone?”, Roberts and Lowe [81] posited that embryonic mortality is the rule rather than the exception, occurring at a rate of ~ 80%. Highly cited reviews over subsequent decades offered qualitative support for this conclusion, estimating rates of 60–70% [82, 83]. Current evidence suggests that a high rate of preclinical losses, not failure of conception, is the main cause of low fecundity of humans, largely driven by chromosome abnormalities [84]. By contrasting aneuploidies observed in arrested and unarrested embryos, our study supports this conclusion and clarifies the role of meiotic and mitotic aneuploidies in early embryonic mortality. Specifically, we show that severe mitotic errors, which frequently arise due to abnormalities in the initial postzygotic cleavage divisions, are the primary cause of mortality among IVF embryos. These data may help guide patients and providers about the average proportions and causes of preimplantation embryo loss. Together, our results suggest that the transition from the cleavage to the blastocyst stage may act as a strong bottleneck wherein numerous aneuploid embryos are eliminated prior to implantation. The timing of this bottleneck is consistent with the increasing reliance on embryonic gene expression as development proceeds through the cleavage, late morula, and early blastocyst stages [85–88].

### Supplementary Information

**Additional file 1:** **Figures S1-S11.** Presenting supplemental results from PGT-A and time-lapse analysis of arrested and developing embryos.

**Additional file 2:** **Tables S1-S11.** Presenting supplemental results from PGT-A and time-lapse analysis of arrested and developing embryos.

## Data Availability

De-identified chromosome-level aneuploidy calls and time-lapse results for all tested embryos are available on Zenodo [89], along with de-identified metadata for the corresponding patients and cycles. Code necessary for reproducing all analyses and figures is archived alongside the data on Zenodo [89].
