# Supplementary material for: Meiotic and mitotic aneuploidies drive arrest of in vitro fertilized human preimplantation embryos
Source: Genome Med. 2023 Oct 2;15:77. doi: 10.1186/s13073-023-01231-1 (PMC10544495; doi:10.1186/s13073-023-01231-1)
Supplement: Supplementary file 1 — Additional file 1: Figures S1-S11. Presenting supplemental results from PGT-A and time-lapse analysis of arrested and developing embryos. [file 13073_2023_1231_MOESM1_ESM.pdf]

Supplementary Figures

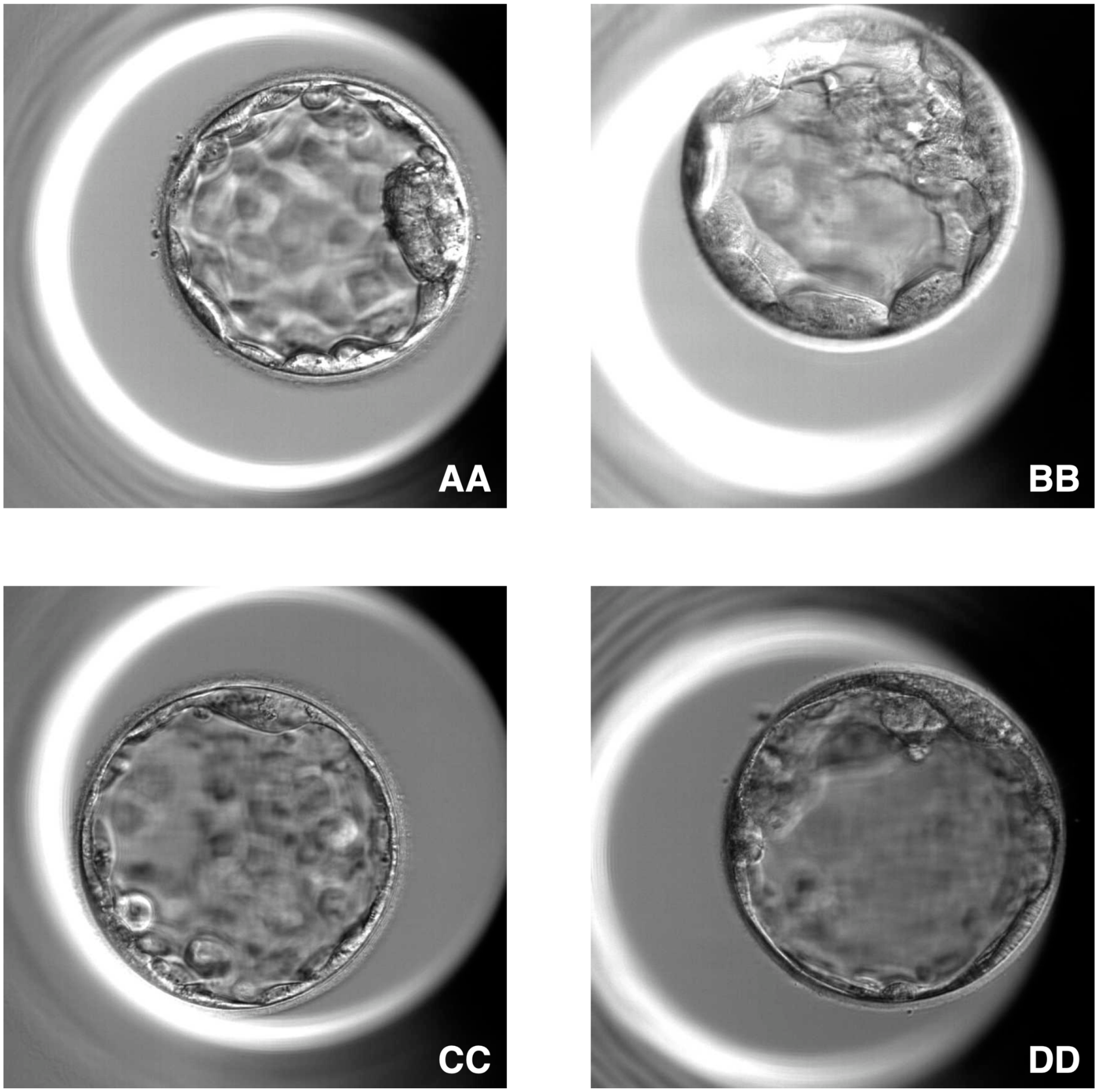

Figure S1. Examples of embryos of various grades as determined according to Alpha/ESHRE (2011) guidelines. Embryos with grades AA, BB, CC, and DD are depicted.

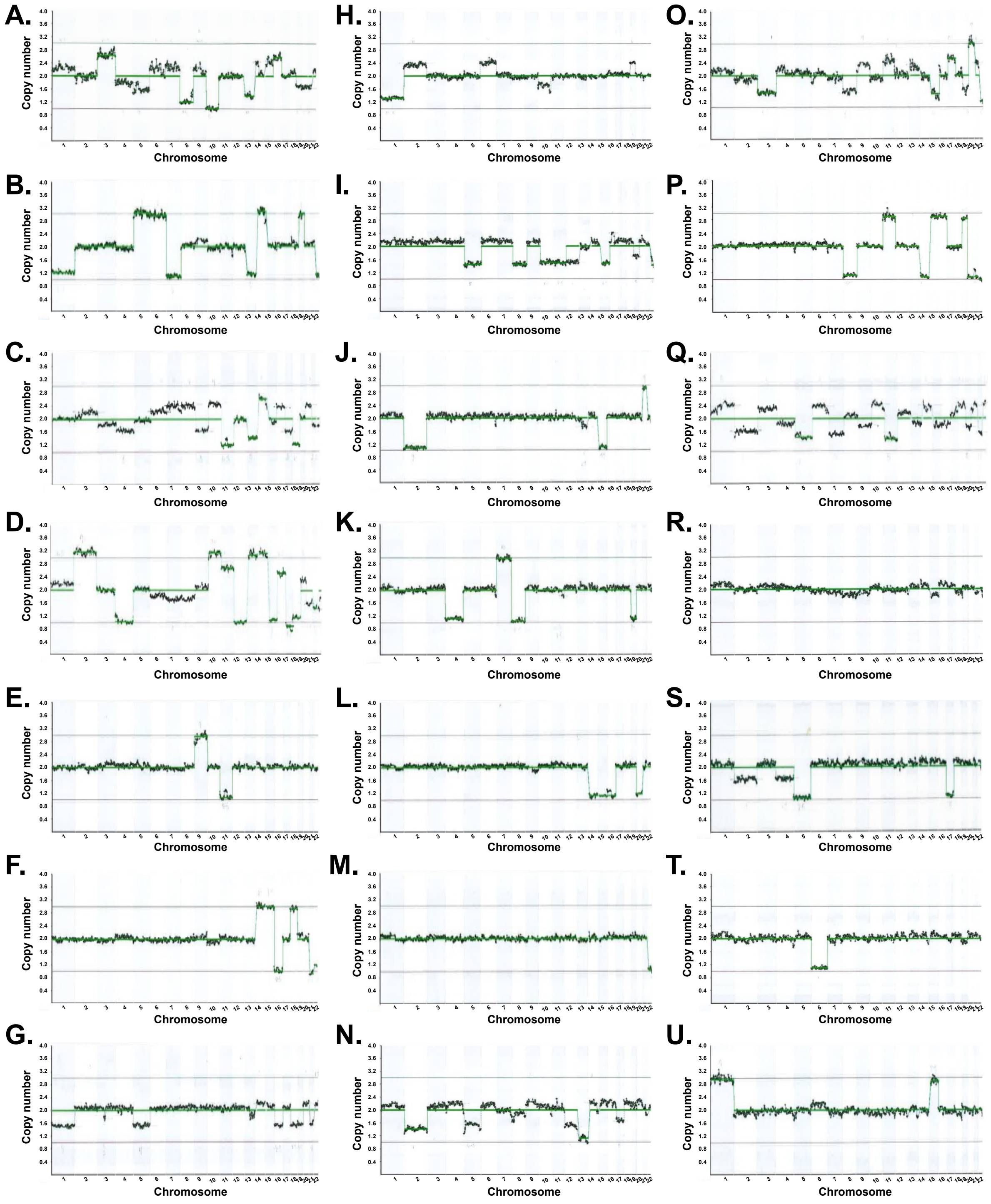

Figure S2. Representative examples of PGT-A results from which chromosome copy number measurements were obtained. Chromosomes are arranged along the X-axes, while inferred copy numbers are depicted on the Y-axes. Upper and lower bounding lines (gray) indicate expectations for full (i.e., putative meiotic) trisomy and monosomy, respectively, while the mid-line indicates expectations for diploidy. Corresponding cell division patterns and survival status

are indicated as follows. **A.** Multipolar first division; 1-4-8 cells; mid arrest. **B.** Normal division; 1-2-4 cells; mid arrest. **C.** Tripolar first division; 1-3-6 cells; early arrest. **D.** Abnormal first division; 1-3-6 cells; early arrest. **E.** No division; 1 cell; early arrest. **F.** Abnormal first division; 1-3-6 cells; mid arrest. **G.** Abnormal first division; 1-4-8 cells; mid arrest. **H.** Tripolar first division; precocious second division; 1-4 cells; early arrest. **I.** Tripolar first division; 1-3-6 cells; mid arrest. **J.** Abnormal second division; 1-2-7 cells; mid arrest. **K.** Abnormal first division; 1-3-10 cells; late arrest. **L.** Abnormal first division; 1-3-9 cells; mid arrest. **M.** Normal division; 1-2-4 cells; late arrest. **N.** Tripolar first division; 1-3-6 cells; late arrest (with cells excluded at compaction). **O.** Abnormal first division; 1-4 cells; mid arrest. **P.** Normal division; 1-2-4 cells; late arrest. **Q.** Abnormal first and second division; 1-4-7 cells; early arrest. **R.** Day-6 expanded blastocyst; 4DD. **S.** Day-6 expanded blastocyst; 4DD. **T.** Day-6 expanded blastocyst; 4DD. **U.** Day-7 expanded blastocyst; 4DD.

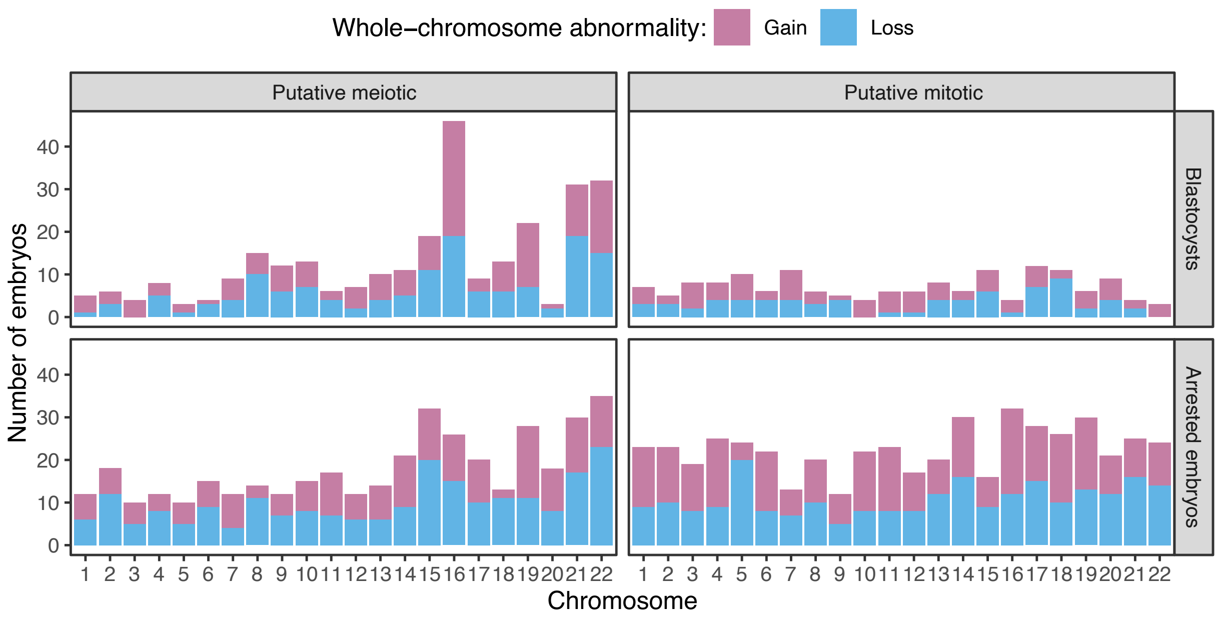

Figure S3. Chromosome-specific counts of putative meiotic (i.e., full copy number change) and putative mitotic (i.e., intermediate copy number change) whole-chromosome gains and losses observed in blastocysts and arrested embryos as determined with PGT-A and restricted to IVF cases where all blastocysts and arrested embryos were tested. Only autosomes are depicted, as distinguishing meiotic and mitotic origins of aneuploidies affecting sex chromosomes poses unique challenges (see Methods).

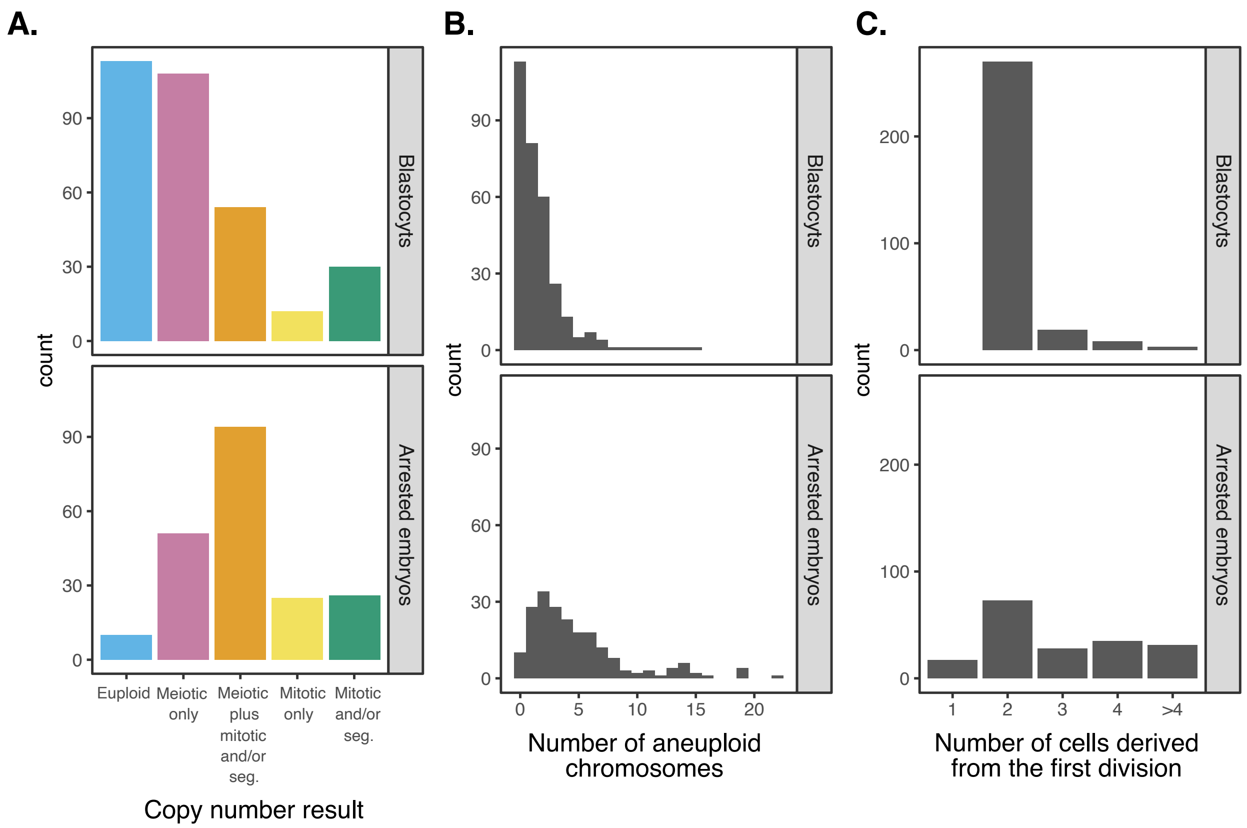

Figure S4. Characteristics of blastocysts and arrested embryos restricted to IVF cases where all blastocysts and arrested embryos were tested. **A.** Counts of arrested embryos versus developing blastocysts, stratified by PGT-A copy number result category. **B.** Counts of arrested embryos versus developing blastocysts, stratified by total number of aneuploid chromosomes. **C.** Counts of arrested embryos versus developing blastocysts, stratified by number of cells present after the first mitotic division.

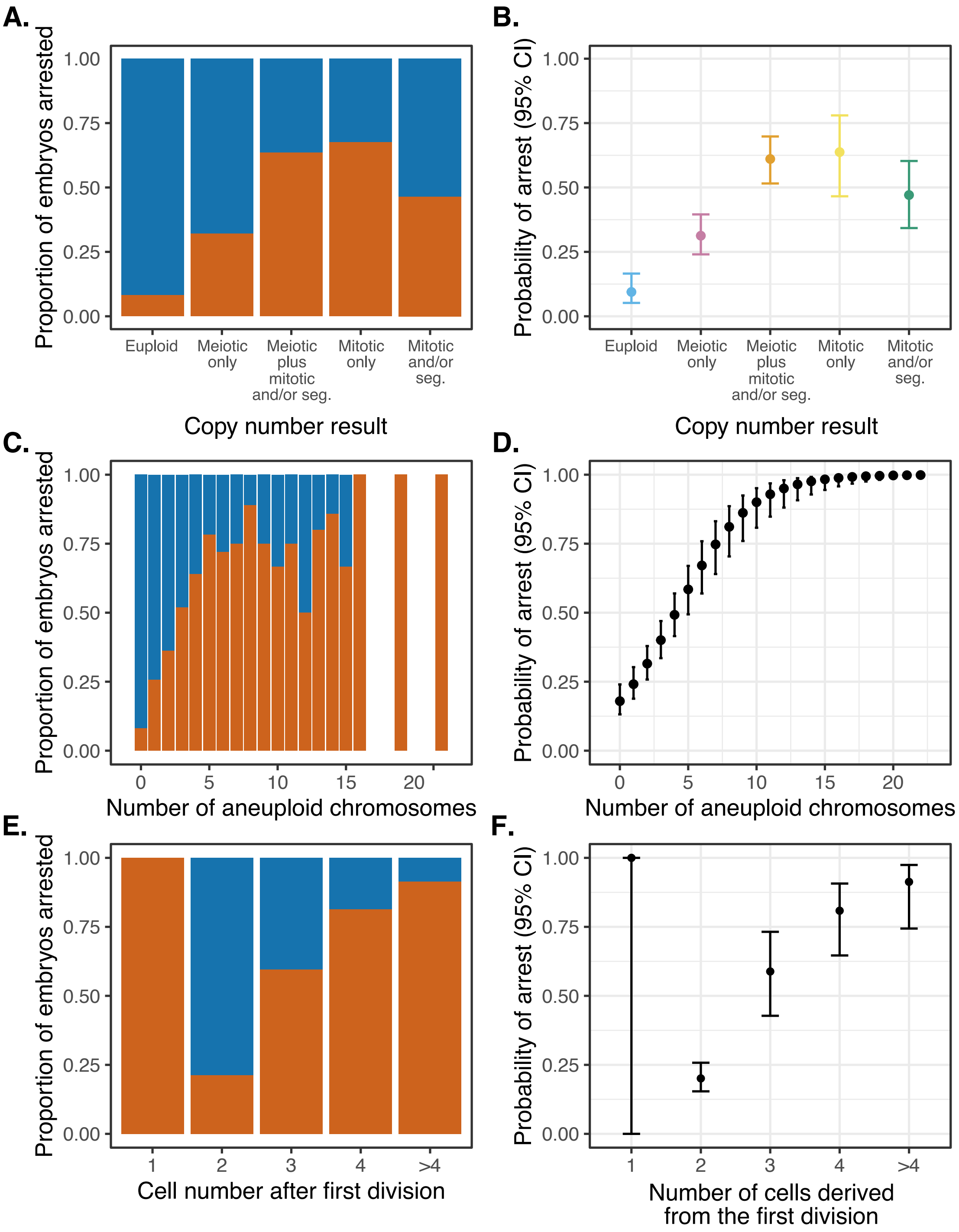

Figure S5. Data (left panels) and statistical modeling (right panels) of the proportion/probability of embryo arrest, stratifying on various patterns of chromosome copy number or cell division. Analyses are identical to those presented in Fig. 4 but restricted to IVF cases where all blastocysts and arrested embryos were tested. Error bars denote 95% confidence intervals of estimates. **A.** Proportion of embryos arrested (red) versus unarrested (blue), stratifying on chromosome copy number pattern, as assessed by PGT-A. **B.** Statistical modeling of the data

from panel A. **C.** Proportion of embryos arrested (red) versus unarrested (blue), stratifying on the number of aneuploid chromosomes. **D.** Statistical modeling of data from panel C. **E.** Proportion of embryos arrested (red) versus unarrested (blue), stratifying on the number of cells observed after the first mitotic division, where 2 is normal. **F.** Statistical modeling of data from panel E.

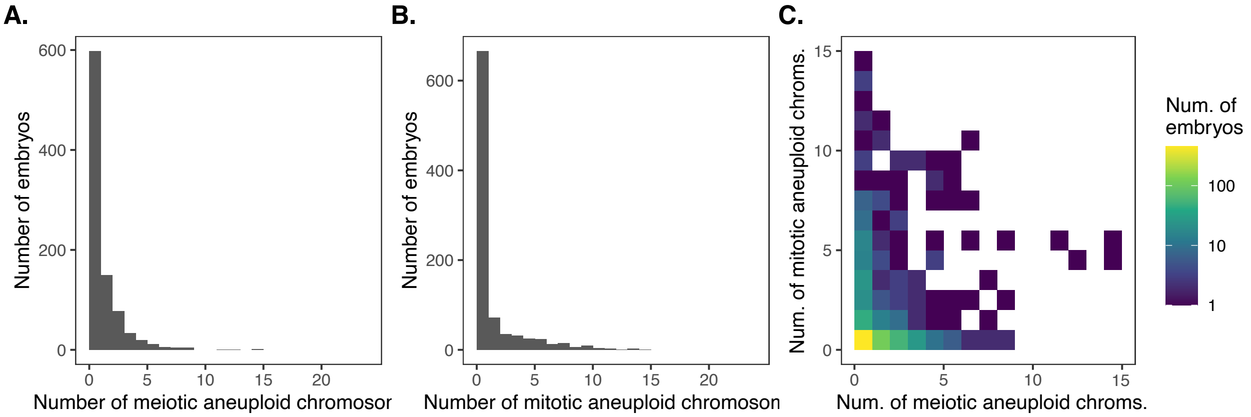

Figure S6. Number of chromosomes affected by meiotic versus mitotic aneuploidy per embryo. **A.** Distribution of number of chromosomes affected with meiotic aneuploidy per embryo. **B.** Distribution of number of chromosomes affected with meiotic aneuploidy per embryo. **C.** Heatmap depicting incidence of meiotic and mitotic aneuploidy affecting different chromosomes of the same embryo.

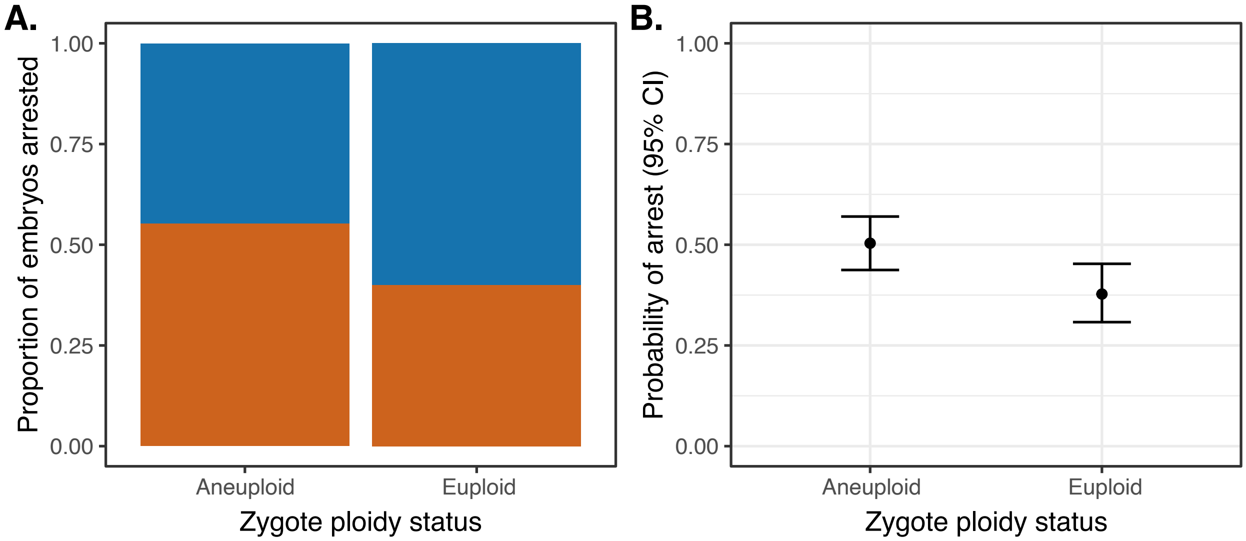

Figure S7. Probability of arrest of embryos derived from euploid zygotes. **A.** Proportion of embryos arrested (red) versus unarrested (blue), stratifying on inferred zygote ploidy status (i.e., presence or absence of putative meiotic aneuploidy, combining the "Meiotic only" and "Meiotic plus mitotic and/or seg." categories from Figure 4). **B.** Statistical modeling of the data from panel A.

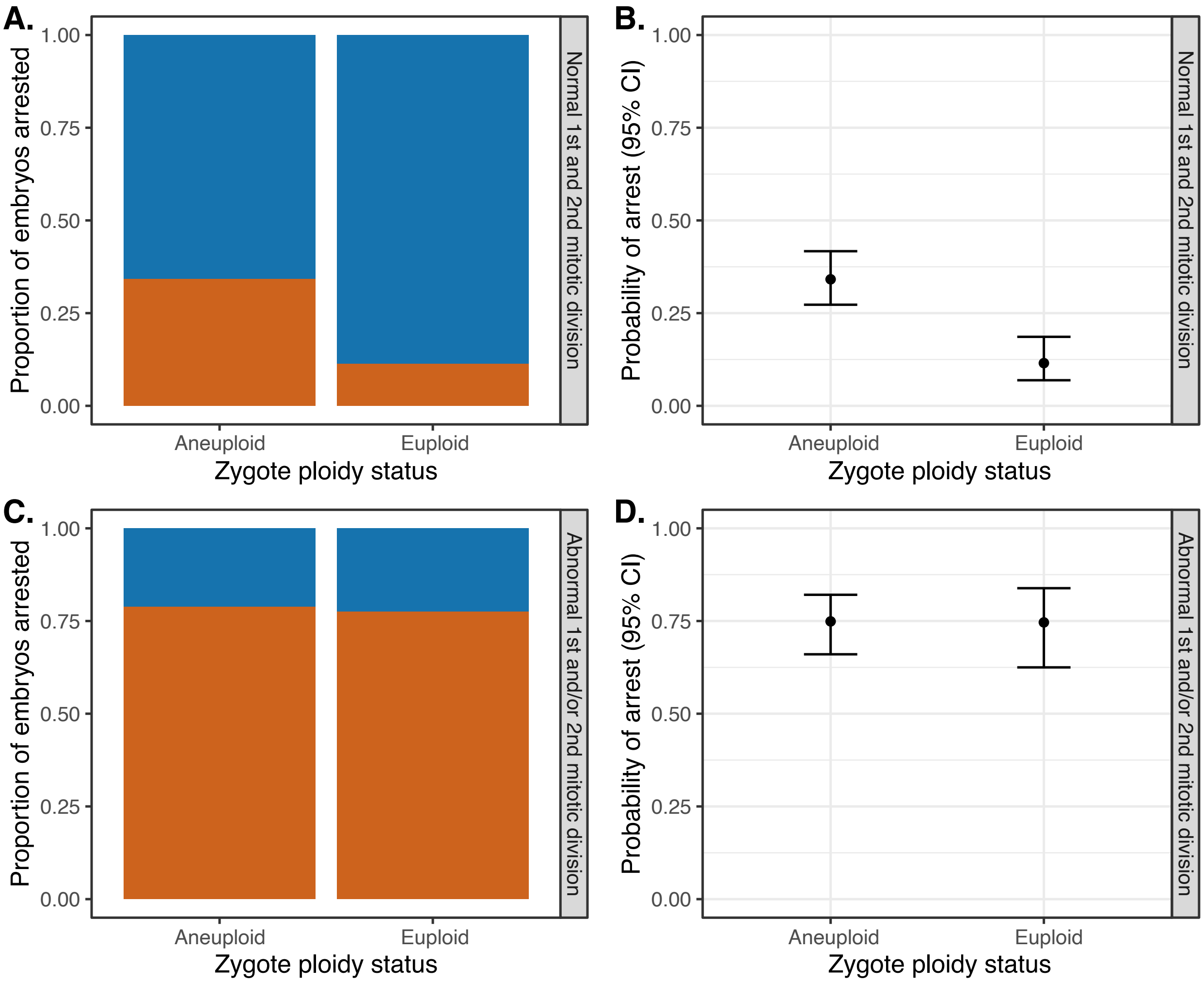

Figure S8. Probability of arrest of embryos derived from euploid zygotes, conditional on outcomes of initial cell divisions. **A.** Proportion of normally dividing embryos arrested (red) versus unarrested (blue), stratifying on inferred zygote ploidy status (i.e., presence or absence of putative meiotic aneuploidy, combining the "Meiotic only" and "Meiotic plus mitotic and/or seg." categories from Figure 4). **B.** Statistical modeling of the data from panel A. **C.** Proportion of abnormally dividing embryos arrested (red) versus unarrested (blue), stratifying on inferred zygote ploidy status. **D.** Statistical modeling of the data from panel C.

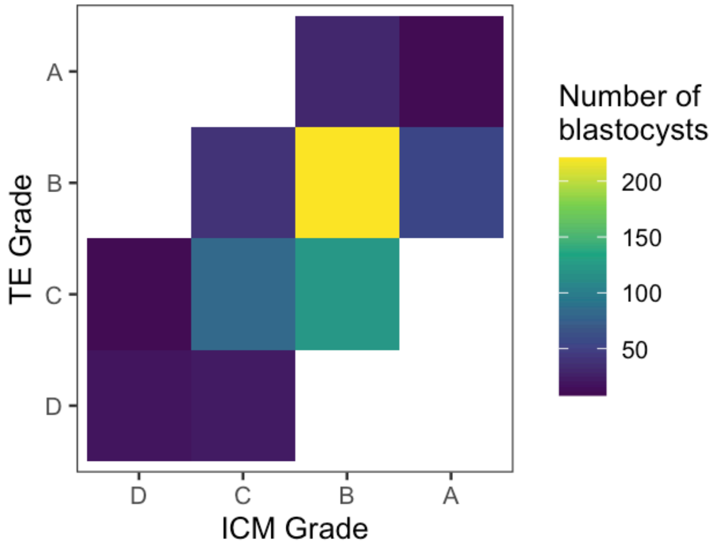

Figure S9. Numbers of embryos with various combinations of TE and ICM morphological grades.

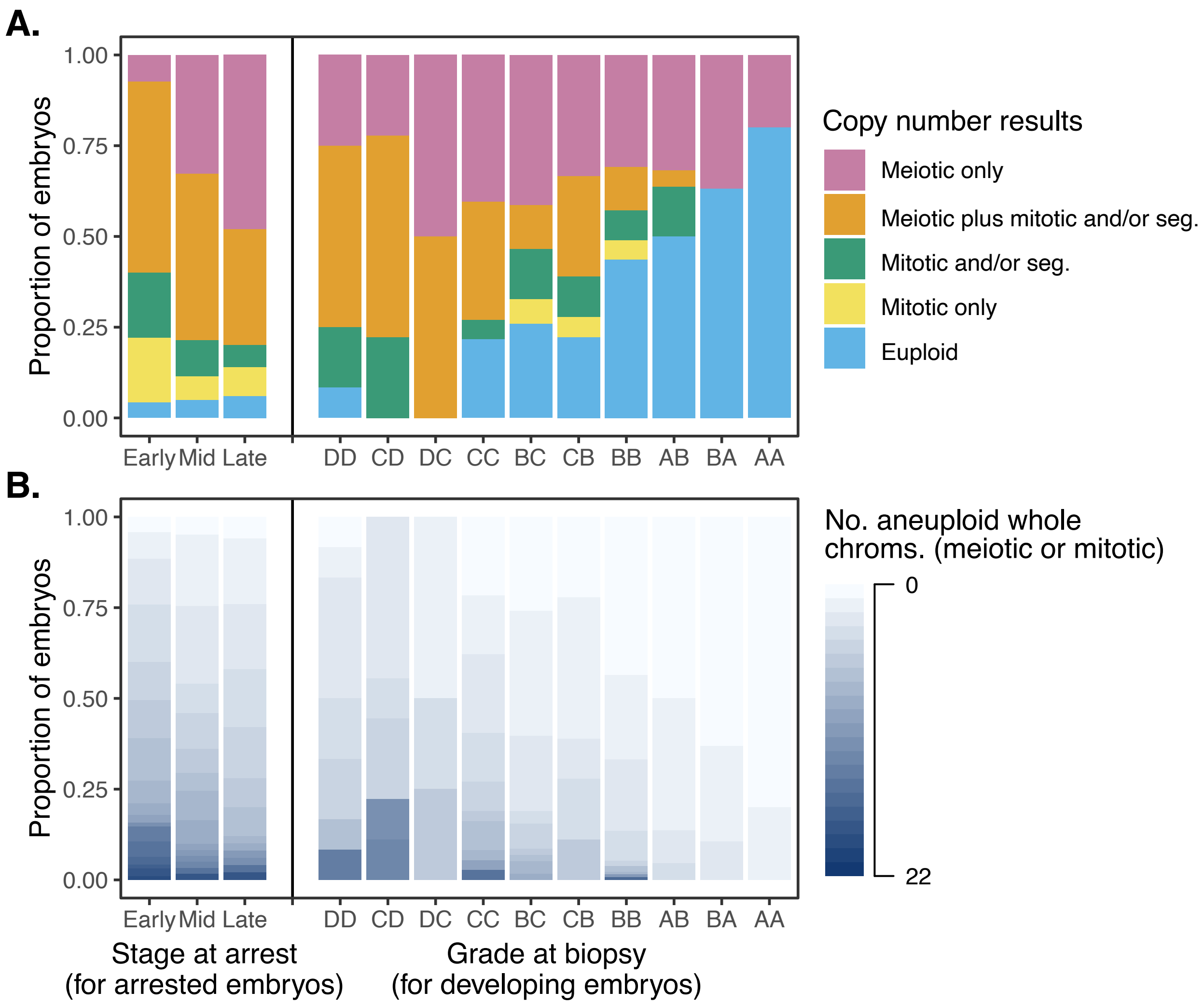

Figure S10. Chromosome copy number results (as assessed via PGT-A) across all tested embryos, stratifying by stage at arrest (see Methods for description of arrested embryos) or morphological grade (for embryos that formed blastocysts). Analyses are identical to those presented in Fig. 5 but restricted to IVF cases where all blastocysts and arrested embryos were tested. ICM grade is listed first, and TE grade is listed second. **A.** Copy number results assigned to categories, as described in Table 1. **B.** Copy number results depicted as counts of aneuploid chromosomes.

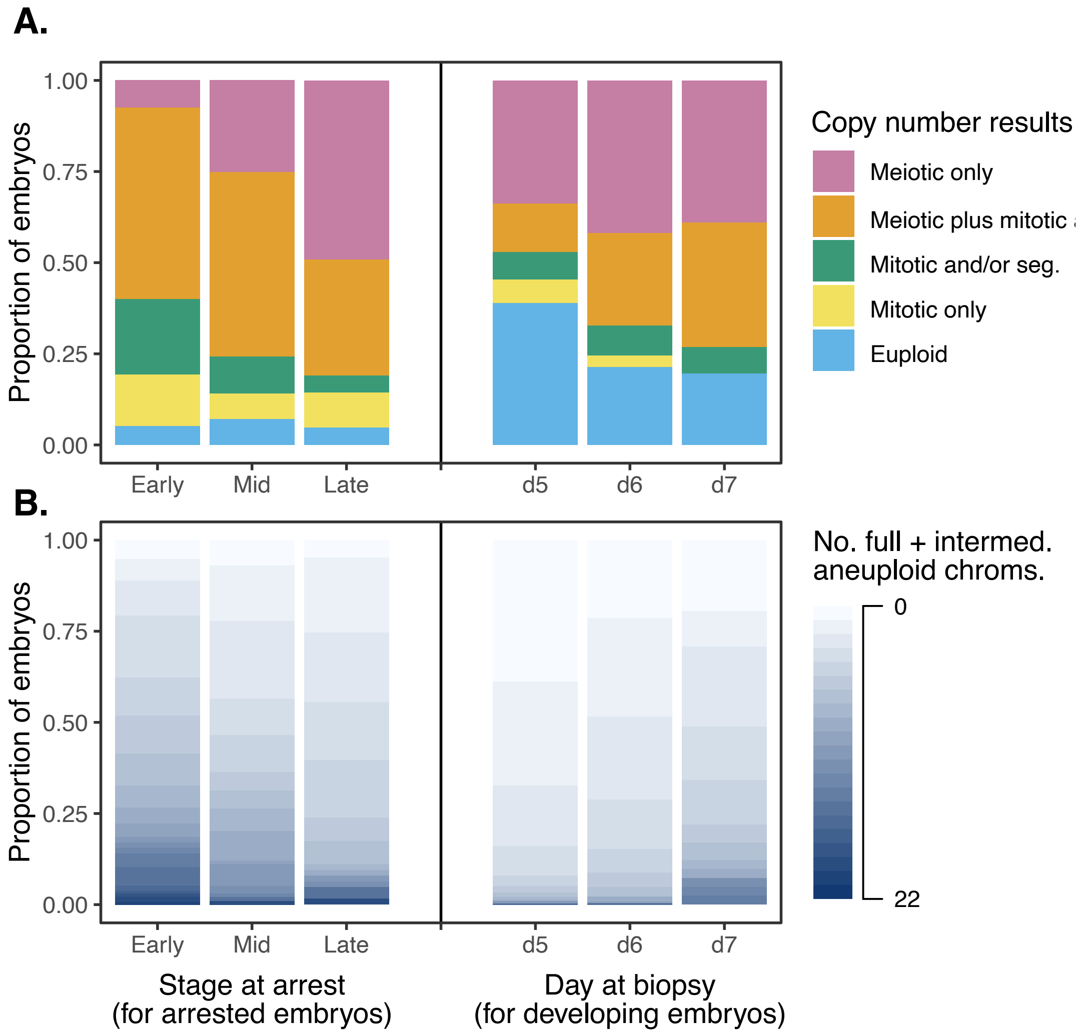

Figure S11. Chromosome copy number results (as assessed via PGT-A) across all tested embryos, stratifying by stage at arrest (see Methods) or day of biopsy (for embryos that formed blastocysts). **A.** Copy number results assigned to categories, as described in Table 1. **B.** Copy number results depicted as counts of aneuploid chromosomes.
