# Supplementary material for: Meiotic and mitotic aneuploidies drive arrest of in vitro fertilized human preimplantation embryos
Source: Genome Med. 2023 Oct 2;15:77. doi: 10.1186/s13073-023-01231-1 (PMC10544495; doi:10.1186/s13073-023-01231-1)
Supplement: Supplementary file 2 — Additional file 2: Tables S1-S11. Presenting supplemental results from PGT-A and time-lapse analysis of arrested and developing embryos. [file 13073_2023_1231_MOESM2_ESM.pdf]

Supplementary Tables

ICM	TE
A : ICM prominent, easily seen, tightly adhered compacted cells	A : Continuous layer of small identical cells
B : ICM less prominent (cells appear compacted and larger in size, loosely adhered)	B : Fewer cells with gaps, not continuous
C : Very few cells visible (cells similar to TE)	C : Fewer small cells with large cells, not continuous
D : No visible cells or visible cells are degenerate or necrotic	D : Sparse cells, large/flat/degenerate

Table S1. A description of the embryo grading scheme employed in our study, which was obtained from the ACE/NEQAS guidelines and is aligned with Alpha/ESHRE recommendations (Alpha/ESHRE, 2011).

		Total counts			Counts per patient		
**Maternal age**	**n**	**2PN**	**Bs**	**Arrested**	**2PN**	**Bs**	**Arrested**
**30**	1	5	3	2	5.0	3.0	2.0
**31**	3	29	24	5	9.7	8.0	1.7
**32**	4	14	8	6	3.5	2.0	1.5
**33**	4	18	8	10	4.5	2.0	2.5
**34**	9	63	42	21	7.0	4.6	2.3
**35**	11	88	47	41	8.0	4.2	3.7
**36**	8	72	40	32	9.0	5.0	4.0
**37**	10	67	42	25	6.7	4.2	2.5
**38**	12	119	61	58	9.9	5.1	4.8
**39**	14	82	40	42	5.9	2.9	3.0
**40**	19	137	86	51	7.2	4.5	2.7
**41**	16	103	56	47	6.4	3.5	2.9
**42**	23	194	91	103	8.4	4.0	4.5
**43**	18	112	48	64	6.2	2.7	3.6
**44**	9	65	21	44	7.2	2.3	4.9
**45**	4	12	5	7	3.0	1.3	1.8
**Total**	165	1180	622	558	7.1	3.8	3.4

Table S2. Maternal age versus the number of dipronuclear (2PN) embryos developing to the blastocyst (Bs) stage. Data exclude 22 cycles where all embryos arrested and were not tested by PGT-A (a total of 52 2PN zygotes)

	No meiotic aneuploidy	Meiotic aneuploidy
**No mitotic aneuploidy**	256	339
**Mitotic aneuploidy**	114	200

Table S3. Number of embryos with meiotic and mitotic aneuploidies, as well as co-occurrence of meiotic and mitotic aneuploidies on different chromosomes of the same samples.

	No meiotic aneuploidy	Meiotic aneuploidy
**No mitotic aneuploidy**	225	257
**Mitotic aneuploidy**	47	83

Table S4. Number of normally fertilized blastocyst-stage embryo biopsies with meiotic and mitotic aneuploidies, as well as co-occurrence of meiotic and mitotic aneuploidies on different chromosomes of the same samples.

	Developed to blastocyst	Arrested
**No mitotic aneuploidy**	482	130
**Mitotic aneuploidy**	113	184

Table S5. Number of embryos with and without mitotic aneuploidy that did versus did not arrest.

	Developed to blastocyst	Arrested
**No meiotic aneuploidy**	272	340
**Meiotic aneuploidy**	98	199

Table S6. Number of embryos with and without meiotic aneuploidy that did versus did not arrest.

Type of first division	n	Rate of aneuploidy	Estimated probability of arrest (95% CI)	Type of second division	n	Rate of aneuploidy	Estimated probability of arrest (95% CI)
**Normal**	621	72.0%	28.1%<br>(22.2% - 34.8%)	**Normal**	540	70.2%	22.7% (17.7% - 28.7%)
				**Multipolar**	40	82.5%	69.6% (46.8% - 85.7%)
				**Precocious**	27	81.5%	72.3% (48.4% - 87.9%)
				**Failed**	12	91.7%	59.0% (27.7% - 84.5%)
				**Reverse**	2	100%	100% (0% - 100%)
**Precocious**	171	86.5%	76.8%<br>(67.1% - 84.3%)	**Normal**	53	81.1%	45.5% (30.3% - 61.6%)
				**Precocious**	14	85.7%	91.7% (54.4% - 99.0%)
				**Failed**	10	80%	26.4% (5.9% - 67.3%)
				**Multipolar**	7	85.7%	100% (0% - 100%)
				**Reverse**	1	100%	100% (0% - 100%)
				**Degenerate**	1	100%	100% (0% - 100%)
**Multipolar**	29	93.1%	84.3%<br>(61.3% - 94.8%)	**Normal**	16	87.5%	77.9% (46.4% - 93.5%)
				**Precocious**	2	100%	100% (0% - 100%)
				**Reverse**	2	100%	100% (0% - 100%)
				**Multipolar**	1	100%	100% (0% - 100%)
				**Failed**	1	100%	100% (0% - 100%)
**Failed**	22	100%	100%<br>(0% - 100%)	**Failed**	8	100%	100% (0% - 100%)
				**Normal**	7	100%	100% (0% - 100%)
				**Precocious**	4	100%	100% (0% - 100%)
				**Multipolar**	3	100%	100% (0% - 100%)

Table S7. Outcomes of first and second cleavage divisions as measured from time-lapse data, along with associated rates of aneuploidy (any form) and estimated probabilities of arrest. Note that in cases of an abnormal first division, the type of abnormal second division was not always possible to classify, so the total sample size for the second division is less than that of the first division. "Multipolar" cleavage refers to the direct cleavage of the zygote (or a daughter cell) into three or more cells. "Precocious" cleavage refers to a rapid division pattern where the zygote (or a daughter cell) undergoes a normal 1→2 cell cleavage, followed by a subsequent premature division to produce 3 or more blastomeres. "Reverse" cleavage refers to the resorption of blastomeres after cytokinesis. "Failed" cleavage refers to multiple rounds of karyokinesis without cytokinesis.

	Divided normally	Divided abnormally
**No mitotic aneuploidy**	412	148
**Mitotic aneuploidy**	127	156

Table S8. Number of embryos with and without mitotic aneuploidy did versus did not divide normally in the first two cell divisions.

	Developed to blastocyst	Arrested
**No meiotic aneuploidy**	235	119
**Meiotic aneuploidy**	304	185

Table S9. Number of embryos with and without meiotic aneuploidy did versus did not divide normally in the first two cell divisions.

	Divided normally	Divided abnormally
**Developed to blastocyst**	463	109
**Arrested**	76	195

Table S10. Number of embryos that divided normally versus abnormally that did versus did not arrest.

	Divided normally	Divided abnormally
**Developed to blastocyst**	157	25
**Arrested**	4	13

Table S11. Number of embryos that divided normally versus abnormally that did versus did not arrest, conditioning on euploidy.
